# Purtscher’s retinopathy and renal cortical necrosis: two rare vaso-occlusive complications in a patient with acute pancreatitis: a case report

**DOI:** 10.1186/s13256-016-1111-4

**Published:** 2016-11-15

**Authors:** Wasim Md. Mohosin Ul Haque, Mehruba Alam Ananna, Hasna Fahmima Haque, Muhammad Abdur Rahim, Tabassum Samad, Sarwar Iqbal

**Affiliations:** 1 Department of Nephrology and Dialysis, Bangladesh Institute of Research and Rehabilitation in Diabetes, Endocrine and Metabolic Disorders (BIRDEM) General Hospital, Shahbag, Dhaka, 1000 Bangladesh; 2 Department of Internal Medicine, Bangladesh Institute of Research and Rehabilitation in Diabetes, Endocrine and Metabolic Disorders (BIRDEM) General Hospital, Shahbag, Dhaka, 1000 Bangladesh

**Keywords:** Acute kidney injury, Acute pancreatitis, Blindness, Purtscher’s retinopathy, Renal cortical necrosis, Case report

## Abstract

**Background:**

Purtscher’s retinopathy and renal cortical necrosis are two rare vaso-occlusive complications of acute pancreatitis. Purtscher’s retinopathy causes sudden impairment of vision, which was first reported in a patient with head trauma. Subsequently, it was also reported as a complication of acute pancreatitis and few other clinical conditions. Acute pancreatitis also rarely causes renal cortical necrosis leading to acute kidney injury. However, the simultaneous presence of both complications is rarely reported.

**Case presentation:**

A 20-year-old Bengali man presented to our hospital with a history of acute upper abdominal pain, vomiting, anuria, and disorientation. He was ultimately found to have bilateral complete blindness due to Purtscher’s retinopathy and acute kidney injury due to renal cortical necrosis, as sequelae of acute pancreatitis. He became dialysis-dependent, his vision did not recover, and he died 16 months after diagnosis.

**Conclusions:**

This case highlights Purtscher’s retinopathy and renal cortical necrosis might be considered as a recognized pair complication of acute pancreatitis.

## Background

Purtscher’s retinopathy is a rare cause of sudden reduction of vision caused by ischemia involving the posterior pole of one or both eyes. The annual incidence of symptomatic Purtscher’s retinopathy is estimated to be 0.24 cases per 1 million population per year [1]. It was first described by Otmar Purtscher in 1910 in a patient with head trauma [2]. Subsequently, a similar retinal clinical picture was found in a few other conditions, including systemic lupus erythematosus, chronic kidney disease, following valsalva maneuver, road traffic accidents with fractured long bone, chest compression, and acute pancreatitis [3–9]. Acute pancreatitis also very rarely causes renal cortical necrosis leading to acute kidney injury (AKI) [10, 11]. Acute cortical necrosis occurs in 1.9–2 % of cases of AKI in developed countries and more frequently (6–7 %) in developing countries [12]. In most of the cases, it is due to obstetric complications [13–16]. There are some isolated case reports of acute pancreatitis with Purtscher’s retinopathy [4, 17–23] and acute pancreatitis with renal cortical necrosis [10, 11, 24–29]. To our knowledge, the simultaneous presence of these two conditions is extremely rare; to date, only one case has been reported [25]. In this report, we describe another such case in a patient who presented with sudden bilateral complete blindness due to Purtscher’s retinopathy and AKI due to renal cortical necrosis following acute pancreatitis.

## Case presentation

A 20-year-old previously healthy Bengali man presented to our hospital with anuria and features of uremic encephalopathy. Ten days prior to this presentation, he had experienced severe upper abdominal pain and vomiting, and he had been treated in a primary care facility for having a case of acute pancreatitis. His initial symptoms improved; however, he gradually became anuric and disoriented. Then he was transferred to our hospital for further management.

At presentation to our hospital, he was severely agitated, restless, and disoriented. He was tachypneic with acidotic breath. Mild pedal edema was present; however, his jugular venous pressure was not raised. His pulse was 112 beats/minute, his blood pressure was 140/90 mmHg, and his body temperature was 98 °F. Signs of meningeal irritation were absent, and his plantar response was bilaterally extensor. His fundus could not be evaluated, and examination of his other systems was unremarkable.

**Fig. 1:**
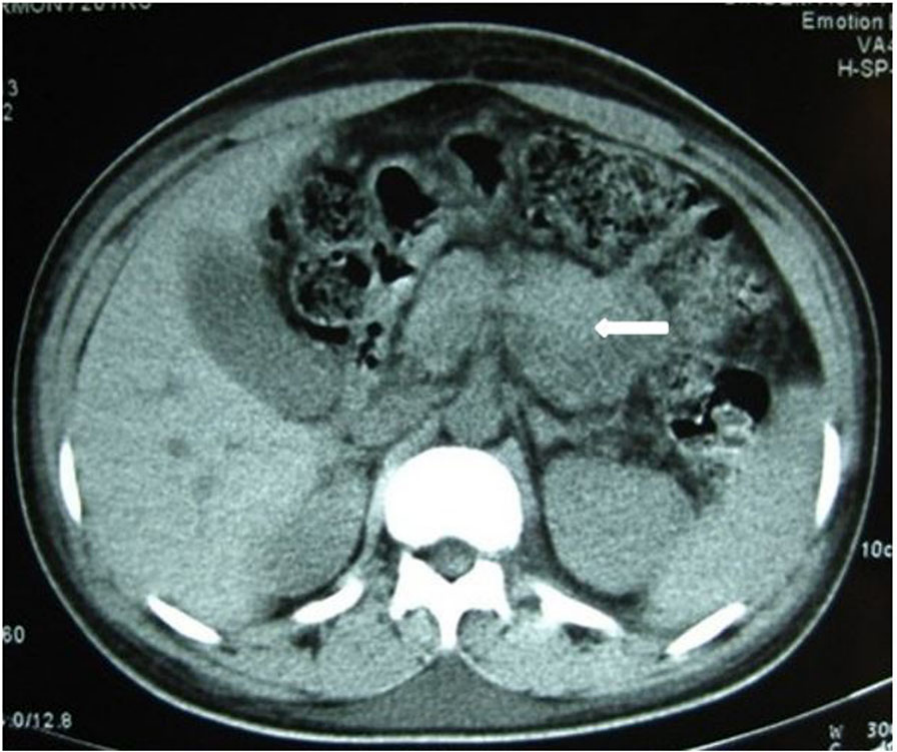
Non-contrast-enhanced computed tomography of the patient’s abdomen shows swollen pancreas (*white arrow*) with peripancreatic edema suggestive of acute pancreatitis

His laboratory parameters showed features of renal dysfunction (serum creatinine 13 mg/dl, serum urea 293 mg/dl), raised pancreatic enzymes (serum amylase 249 U/L [reference up to 100 U/L], serum lipase 227 U/L [reference 13–60 U/L), normal liver function tests (serum bilirubin 0.9 mg/dl, alanine aminotransferase 38 U/L, aspartate aminotransferase 35 U/L, alkaline phosphatase 122 U/L, serum albumin 37 g/L), normal potassium (5.1 mmol/L), normal bicarbonate (19 mmol/L), and normal triglycerides (173 mg/dl). His serological markers, including antinuclear antibodies, cytoplasmic antineutrophil cytoplasmic antibodies, perinuclear antineutrophil cytoplasmic antibodies, C3, and C4, were within normal limits. An ultrasonogram of his whole abdomen was unremarkable, but non-contrast-enhanced computed tomography (CT) findings were suggestive of acute pancreatitis (Fig. 1). His kidneys were unremarkable, however.

**Fig. 2:**
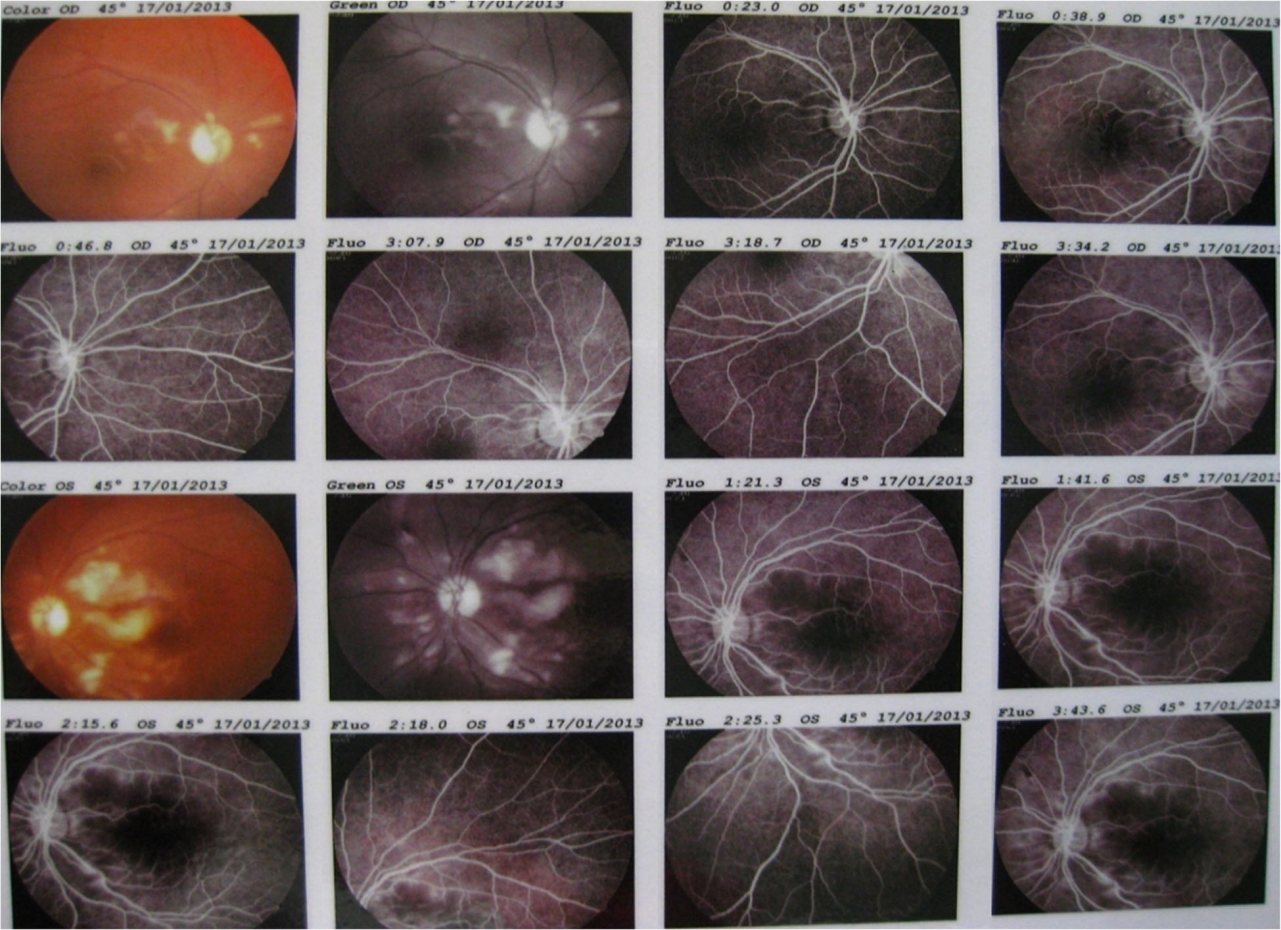
Fundus photographs of both eyes show cotton wool spots at the posterior pole and peripapillary areas. Fluorescein fundus angiography of both eyes (early, middle, and late phases) shows an increased foveal avascular area and a capillary dropout area at the posterior pole

**Fig. 3:**
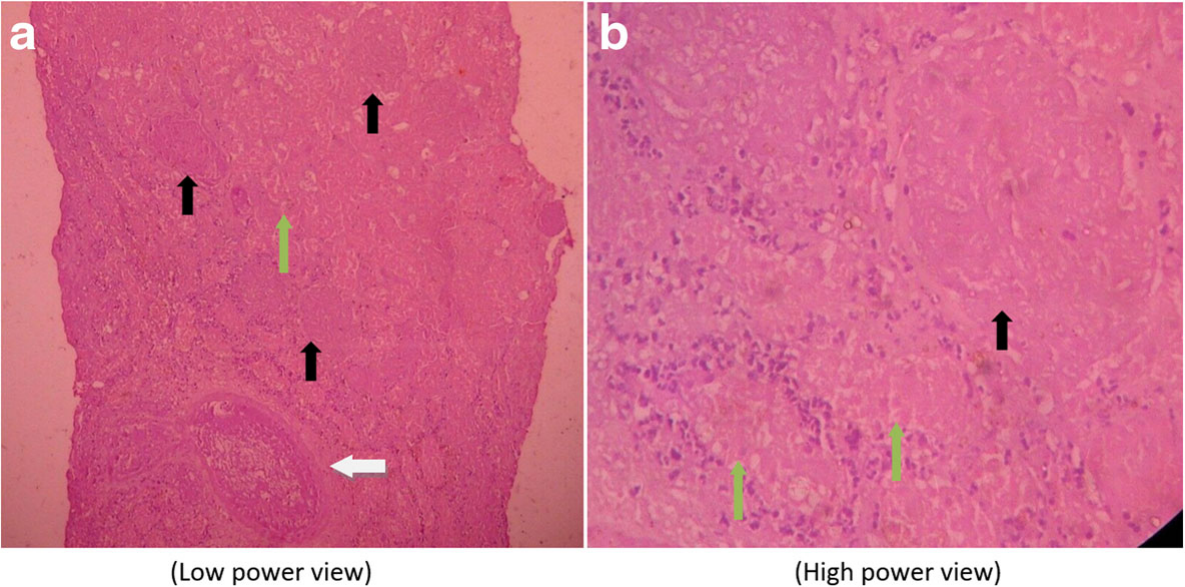
Hematoxylin and eosin-stained sections from core biopsy reveal renal cortex containing few glomeruli and expected number of renal tubules. Glomeruli (*black arrows* in **a** and **b**) and tubules (*green arrows* in **a** and **b**) show extensive coagulation necrosis, with few of the tubular cells having karyolytic nuclei. No viable glomerulus or tubule is discernible; however, the connective tissue framework is preserved. Sections also reveal necrosed blood vessels, including a necrosed and thrombosed intralobular artery (*white arrow* in **a**). Neutrophilic infiltrates are also present in the interstitium surrounding necrosed glomeruli and tubules

The patient was managed as having a case of AKI and acute pancreatitis. Urgent hemodialysis was initiated. After he had received two sessions of hemodialysis, his level of consciousness improved, but he complained of profound visual loss. An assessment revealed only perception of light. A funduscopic examination showed retinal whitening and extensive cotton wool exudates as well as Purtscher’s flecken (Fig. 2) compatible with Purtscher’s retinopathy. High-dose parenteral methylprednisolone (1 g intravenously once daily for 3 days) was administered. For evaluation of renal dysfunction, a renal biopsy was done; the histopathological findings were compatible with renal cortical necrosis (Fig. 3).

The patient denied substance abuse or alcohol ingestion. He did not consume any nephrotoxic drugs or herbal products in the recent past. No history suggesting connective tissue diseases was available, nor did he have a history of hypotension throughout the course of his current illness.

The patient was finally diagnosed with acute pancreatitis complicated with renal cortical necrosis leading to AKI and Purtscher’s retinopathy leading to complete bilateral blindness. He died 16 months after his initial presentation as a result of a recurrent attack of acute pancreatitis. Before that, he had been undergoing maintenance hemodialysis; however, his renal function did not recover, though his vision was improved to finger-counting at 2 feet.

## Discussion

Acute pancreatitis in most cases presents in a milder form with prompt recovery. However, in about 20 % of cases, it can present in severe form with local and systemic complications. Acute pancreatitis rarely leads to Purtscher’s retinopathy and renal cortical necrosis [4, 17–23]. Though several mechanisms are described, both can be caused by a common phenomenon, which might have occurred in our patient. The presence of pancreatic proteases in the systemic circulation following acute pancreatitis is the inciting factor. Subsequently, activation of complement cascade leads to the formation of C5a-induced leukocyte, platelet, and fibrin aggregation. This ultimately causes retinal and renal cortical embolization and ischemia, leading to life- and organ-threatening complications, as in our patient [21–23, 30–32]. Endothelin released from damaged vessels also contributes to this process by producing vasospasm, which further aggravates ischemia.

Association of Purtscher’s retinopathy with acute pancreatitis was first described by Inkeles and Walsh in 1975 [4]. Purtscher’s retinopathy clinically manifests as no visual impairment to a varying degree of visual impairment, which in many cases improves spontaneously with time [33]. This retinopathy is characterized by the presence of cotton wool spots and flame-shaped hemorrhages located only in the end arteriolar retinal circulation around the disc and macula, described in 83–92 % of a series of cases [34]. Purtscher flecken (intraretinal whitening with a clear zone on either side of the retinal arterioles, venules, and precapillary arterioles) are considered to be pathognomonic, but it occurs in only 50 % of cases [34]. All of the above-mentioned funduscopic findings were present in our patient (Fig. 2). The outcome of Purtscher’s retinopathy is uncertain; however, in most of the cases, visual acuity improves with time [33, 35]. Resolution of retinal lesions is expected within about 4 months. Male sex and pancreatitis as a cause are usually associated with good prognosis [33]; unfortunately, our patient’s visual acuity did not improve significantly. Considering the beneficial effect of high-dose steroid treatment, as documented in a few case reports [36, 37], we administered intravenous methylprednisolone for 3 consecutive days, which appeared to be ineffective as observed in many other cases [1, 33, 34].

Renal cortical necrosis is an extremely rare complication of acute pancreatitis [10, 11, 24–29]. The lesion is irreversible, leading to total loss of kidney function and end-stage renal disease in severe cases. Anuria is the usual presenting symptom of acute renal cortical necrosis. Prolonged anuria (>4 weeks) in appropriate clinical settings suggests the clinical diagnosis of renal cortical necrosis [12]. Renal hypoperfusion resulting from prolonged shock and direct or indirect endothelial injury can cause renal cortical necrosis. In acute pancreatitis other than hypovolemic shock, the presence of proteolytic enzymes in the systemic circulation indirectly causes endothelial injury and severe spasm of renal microcirculation, leading to diffuse ischemic necrosis of the cortical structures, such as vessels, tubules, and glomeruli [10–12]. In our patient, renal histopathology showed extensive coagulative necrosis of all the cortical structures, including glomeruli, tubules, and vessels (Fig. 3). The tissue from the medulla was viable. Direct immunofluorescence showed no deposition. Though histopathology is the gold standard in diagnosing renal cortical necrosis, it can often be reliably replaced by contrast-enhanced CT. Low attenuation of cortex spearing thin rim of subcapsular cortex and medulla on contrast-enhanced CT scans is a characteristic finding in renal cortical necrosis; however, to minimize the further risk of kidney damage in our patient, we did not perform contrast-enhanced CT [38, 39]. Slater *et al*. described a case of acute pancreatitis complicated with Purtscher’s retinopathy and renal cortical necrosis, similar to our patient’s case [25]. Their patient had no visual impairment; his renal function improved gradually over time; and by 2 years he became dialysis-free. His biopsy showed renal cortical necrosis with patent vessels; however, in our patient, the vessels were also involved in the necrotic process, indicating more extensive damage. Our patient’s fatal outcome may imply the severity of his disease.

Renal outcome in renal cortical necrosis depends on the severity of damage. In patchy lesions, renal function usually recovers, but in cases with diffuse involvement, end-stage renal disease is the norm [12]. However, in certain cases, there may be a slow rise in creatinine clearance, and a gradual gain in renal function has been observed over 1–2 years. The glomerular filtration rate may reach a final plateau level of approximately 20–24 ml/minute [40, 41]. Our patient’s renal impairment did not recover even 16 months after the onset of illness, which might be due to the extensive nature of the damage.

## Conclusions

Because of the paucity of cases, widely accepted, uniform management guidelines for treating Purtscher’s retinopathy and renal cortical necrosis complicating acute pancreatitis are lacking. Cases have been managed in different ways; however, improvement of visual acuity in Purtscher’s retinopathy and partial improvement of renal function in acute cortical necrosis with time are not uncommon. In our patient, the outcome was fatal, which might have been due to the severity of his disease. Though rare, simultaneous occurrence of Purtscher’s retinopathy and renal cortical necrosis might be considered as a recognized paired complication of acute pancreatitis.

## Abbreviations

AKI: Acute kidney injury
CT: Computed tomography
